# Influence on Elastic Wave Propagation Behavior in Polymers Composites: An Analysis of Inflection Phenomena

**DOI:** 10.3390/polym15071680

**Published:** 2023-03-28

**Authors:** Guoqiang Luo, Pu Cheng, Yin Yu, Xiangwei Geng, Yue Zhao, Yulong Xia, Ruizhi Zhang, Qiang Shen

**Affiliations:** 1 Chaozhou Branch of Chemistry and Chemical Engineering Guangdong Laboratory, Chaozhou 521000, China; 2 State Key Lab of Advanced Technology for Materials Synthesis and Processing, Wuhan University of Technology, Wuhan 430070, China; 3 National Key Laboratory of Shock Wave and Detonation Physics, Institute of Fluid Physics, China Academy of Engineering Physics, Mianyang 621900, China

**Keywords:** particle polymer composites, elastic wave, wave attenuation, wavefront

## Abstract

Particulate polymer composites (PPCs) are widely applied under different elastic wave loading conditions in the automobile, aviation, and armor protection industries. This study investigates the elastic wave propagation behavior of a typical PPC, specifically a Cu/poly (methyl methacrylate) (*PMMA*) composite, with a wide range of particle contents (30–65 vol. %) and particle sizes (1–100 μm). The results demonstrate an inflection phenomenon in both the elastic wave velocity and attenuation coefficient with increasing volume content. In addition, the inflection point moves to the direction of low content with the increase in particle size. Notably, the elastic wave velocity, attenuation, and wavefront width significantly increased with the particle size. The inflection phenomenon of elastic wave propagation behavior in PPCs is demonstrated to have resulted from particle interaction using the classical scattering theory and finite element analysis. The particle interaction initially intensified and then reduced with increasing particle content. This study elucidates the underlying mechanism governing the elastic wave propagation behavior of high particle content PPCs and provides guidelines for the design and application of wave-absorbing composites.

## 1. Introduction

A particulate polymer composite (PPC) is an amalgamation of matrix materials and fillers that determines their properties through interactions between the matrix, fillers, and the interstitial region between them [1,2,3,4,5]. Fillers are incorporated into the matrix to augment their resistance to high-frequency waves [6], rendering PPCs appropriate for use in various engineering domains, such as waveguides [7], automotive [8], aviation, defense, and naval structures [9,10,11]. The absorption of elastic waves in PPCs with periodic arrangements of particles has been extensively studied, and the results have demonstrated that PPCs exhibit remarkable properties in absorbing elastic waves [12,13,14,15,16]. This has been substantiated through a combination of experimental and computational studies and the implementation of phononic crystal theory [17,18,19]. In conclusion, PPCs are promising materials for protection against high-frequency waves and demonstrate a wide range of potential applications across multiple engineering fields [20].

The propagation of elastic waves in a particulate polymer composite (PPC) is influenced by several factors, including the concentration of particles, size and distribution, mechanical properties of both the matrix and fillers, and frequency of elastic waves [21,22]. Beltzer [23] proposed a novel approach for examining the propagation of elastic waves in random particle viscoelastic composites, considering the effects of scattering, losses, and Kramers–Kronig relations for calculating attenuation and dispersion. Meysam [24,25] utilized the pulse echo method to investigate aluminum-based nanocomposites and discovered a linear correlation between the velocity of elastic waves and Young’s modulus, as well as between the uniformity of attenuation, dispersion, and porosity. Huang [26] discovered that the velocity of waves decreased and the level of attenuation increased with a higher copper content, confirming the predictions of the Voigt Model. This model is a widely accepted framework used to describe the characteristics of viscoelastic materials. Biwa [27,28,29] used a viscoelastic theoretical model to investigate the scattering and absorption of particles in PPCs and discovered that the predictions were higher than the observed values, owing to the omission of the impact of particle interactions on the total attenuation. This highlights the significance of considering particle interactions and the need for further research in this field.

According to the Voigt model [30], composite materials should exhibit an “inflection point” in the elastic wave velocity and attenuation coefficient when the filler content reaches 50%. However, this phenomenon has received limited attention in previous studies, which have mostly focused on composites with filler content below the “inflection point”. Fu [31] used the pulse echo method to examine the effect of incorporating Ethylene Propylene Diene Monomer (EPDM) into polystyrene (PS) on elastic wave transmission. The results indicated that the velocity and attenuation of the waves varied in accordance with the increase in EPDM content, and a turning point was observed. A similar phenomenon was observed in TiO_2_/Polydimethylsiloxane (PDMS) composites [32]. As the concentration of TiO_2_ increased from 0 to 60 vol. %, the velocity of elastic waves initially declined and then augmented. The study also drew on two theoretical models, the coherent potential approximation model and the Waterman–Truell multiple scattering model, to reinforce the discoveries. However, the exact cause of this phenomenon remains unknown. Therefore, there is a need for further research to better understand the behavior of composite materials beyond this inflection point.

Li [33,34,35,36] observed a commensurate inflection in the propagation of shock waves in aluminum powder/rubber matrix composites. Through a fusion of experiments and simulations, Li investigated the effect of particle size and content on the attenuation of elastic waves in PPCs. The research parsed the respective impacts of matrix absorption, independent particle scattering, and particle interaction on the total elastic wave attenuation. The results indicated that the particle interaction considerably augmented the attenuation coefficient with increasing particle content. This emphasizes the indispensability of considering particle interactions when scrutinizing the attenuation of elastic waves in the PPC. The propagation of elastic waves is intricate, and few studies have proposed a lucid method for appraising the contributions of single scattering and interaction to wave attenuation.

It is well established that particle size exerts a significant influence on the properties of composites [37]. Cheng [38] analyzed the impact of particle content and size on the elastic wave attenuation coefficient in SiO_2_/Polyurea composites and found that both particle number and diameter increased the attenuation of PPC. The results showed that particle scattering was the main cause of enhanced wave attenuation, which was verified by comparing the simulation and theoretical results. Mylavarapu [39] developed a model that considers factors such as the particle size, porosity, and radius ratio to determine the elastic wave attenuation coefficient. Nonetheless, this investigation did not consider the impact of particle interactions because of the small PPC content.

Thus, we crafted Cu/PMMA (polymethyl methacrylate) composites with particle sizes ranging from 1 to 100 μm and contents ranging from 30 to 65 vol. % with the aim of scrutinizing the influence of particle size and content on elastic wave propagation in PPC. Both the pulse echo method and finite element simulation were used to investigate the effect of particle size and content on the elastic wave velocity and attenuation of the PPC.

## 2. Materials and Methods

### 2.1. Materials and Preparation

The PPC were fabricated using copper (Cu) powder and poly (methyl methacrylate) (PMMA) with three particle sizes (1, 10, and 100 μm). Cu powder with particle sizes of 1.32, 6.873, and 155.6 μm was provided by Shanghai Pantian Powder Co., Ltd., China. PMMA (MF 001) and was acquired from Mitsubishi Chemical Polymer Nantong Co., Ltd.; it had a molar mass (Mw) of 110,000 g/mol and density (ρ) of 1.19 g/cm^3^. The glass transition temperature (T_g_) of PMMA was 105.5 °C, which was measured using differential scanning calorimetry (DSC 2500, T.A. Instruments, New Castle, DE, USA).

Cu/PMMA composites were fabricated by blending PMMA and Cu powder using a torque rheometer (XSS 300, Shanghai Kechuang, Shanghai, China). The mixture was retained until the torque curve became steady and was subsequently introduced into a mold for hot pressing at a temperature of 170 °C and pressure of 10–20 MPa using a hot press machine (XLD B 300×300×1 MN, Jinjiuzhou Rubber Machinery Co., Ltd., Qingdao, China). The resulting composite plates were subsequently trimmed to the required dimensions for characterization.

### 2.2. Characterization

#### 2.2.1. Content Testing

Prior to conducting the tests, the samples were sectioned into small fragments, and a 1-g sample was procured for each test. The actual Cu content in the composites was assessed using a synchronous thermal analyzer (STA 449F3, NETCZSCH, Selb, German). To guarantee precision and eliminate extraneous factors, high-purity nitrogen was used during the tests at a flow rate of 150 mL/min. Nitrogen was purged for 20 min before initiating the heating program to create an inert environment. The thermal decomposition was performed at a slow heating rate of 10 °C /min to minimize heat and mass transfer intrusions.

To prevent contamination or damage to the sample and ensure accurate results, the maximum temperature was limited to 900 °C, which is below the melting point of Cu (approximately 1083.4 °C) and well above the decomposition temperature of PMMA (300–400 °C). This ensured complete decomposition of the PMMA and prevented the liquefaction of Cu.

The actual Cu content ($\phi_{A}$) was calculated using Equation (1):

(1) $$\phi_{A}=\frac{\left(\frac{C_{m}}{\rho_{\mathrm{Cu}}}\right)}{\frac{C_{m}}{\rho_{\mathrm{Cu}}}+\frac{1-C_{m}}{\rho_{\mathrm{PMMA}}}}\times 100\%,$$

where $C_{m}$ is the mass fraction of residual material in the sample at 900 °C, $\rho_{\mathrm{Cu}}$ and $\rho_{\mathrm{PMMA}}$ are the densities of Cu (8.924 g/cm^3^) and PMMA (1.18 g/cm^3^), respectively.

#### 2.2.2. Microstructure

A portion of each sample was sectioned for microstructural analysis. The fracture surfaces were adorned with a delicate coating of gold using a sputter coating technique. The morphology of the specimens was then examined using high-resolution field emission scanning electron microscopy (FESEM, Quanta 250, USA). Moreover, the three-dimensional microstructure of the Cu/PMMA composites was explored using an X-ray microscope (Xradia 510 Versa, Oberkochen, Germany).

#### 2.2.3. Elastic Wave Properties Measurements

The analysis of the elastic wave propagation in the composite samples was performed using a high-resolution ultrasonic scanning microscope (WINSAM Vario III, Kraemer Scientific Instruments GmbH, Lerchenweg, Germany) with a sampling frequency of 500 MHz and a bandwidth range of 5 to 500 MHz. The elastic waves were transmitted and received by a transducer (V313-SU, Olympus NDT Corporation, Tokyo, Japan) with a center frequency of 5 MHz.

**Figure 1:**
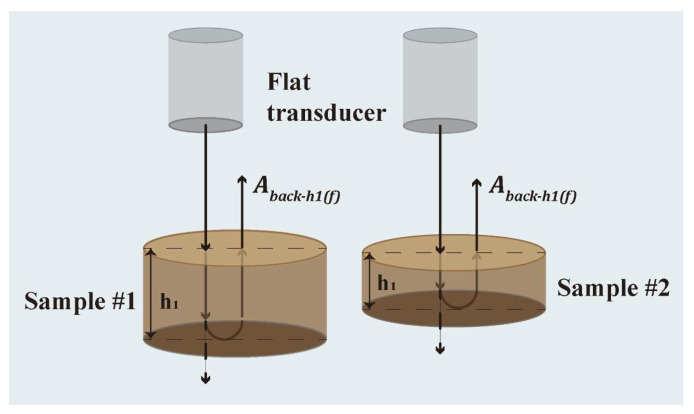
Schematic of an ultrasonic pulse-cho test.

The velocity and attenuation of elastic waves were measured using a pulse-echo experiment with a planar immersion transducer, as shown in Figure 1. During the experiment, a single transducer was used to transmit and receive elastic waves that propagated through the water and solid samples. Samples 1 and 2 with thicknesses h1 and h2, respectively, were prepared for the test. Echo signals were obtained under identical test conditions to eliminate the effect of the medium and interface on the elastic wave. The strength of the received elastic wave ($A_{backh_{1}}\left(f\right)$, $A_{backh_{2}}\left(f\right)$) was calculated from the time-domain signal received by the oscilloscope using Fast Fourier Transform (FFT). The experiment was repeated five times for each sample to ensure repeatability.

(2) $$A_{back-h_{1}}\left(f\right)=A\left(f\right)T_{12}R_{21}T_{21}e^{2ikh_{1}},$$

(3) $$A_{back-h_{2}}\left(f\right)=A\left(f\right)T_{12}R_{21}T_{21}e^{2ikh_{2}},$$

where f is the frequency of the elastic wave, $T_{21}$ is the transmission coefficient of the elastic wave incident from medium 1 to medium 2, $T_{21}$ is the transmission coefficient of the ultrasonic waves incident from medium 2 to medium 1, $R_{21}$ is the reflection coefficient of the ultrasonic waves incident from medium 2 to medium 1, h is the sample thickness, and k is the wave number in the samples.

(4) k=2π/c(f)+iα(f),

where c and α are the wave velocity and the attenuation coefficient of the sample, respectively. The wave velocity and attenuation coefficient were calculated using the following formulas:

(5) $$c\left(f\right)=\frac{2\left(h_{2}-h_{1}\right)w}{arg\left[\frac{A_{back-h_{1}}\left(f\right)}{A_{back-h_{2}}\left(f\right)}\right]+2N\pi },$$

(6) $$\alpha \left(f\right)=\frac{1}{2\left(h_{2}-h_{1}\right)}ln\left|\frac{A_{back-h_{1}}\left(f\right)}{A_{back-h_{2}}\left(f\right)}\right|,$$

where 2Nπ is the phase correction constant.

### 2.3. FEM Simulation

**Figure 2:**
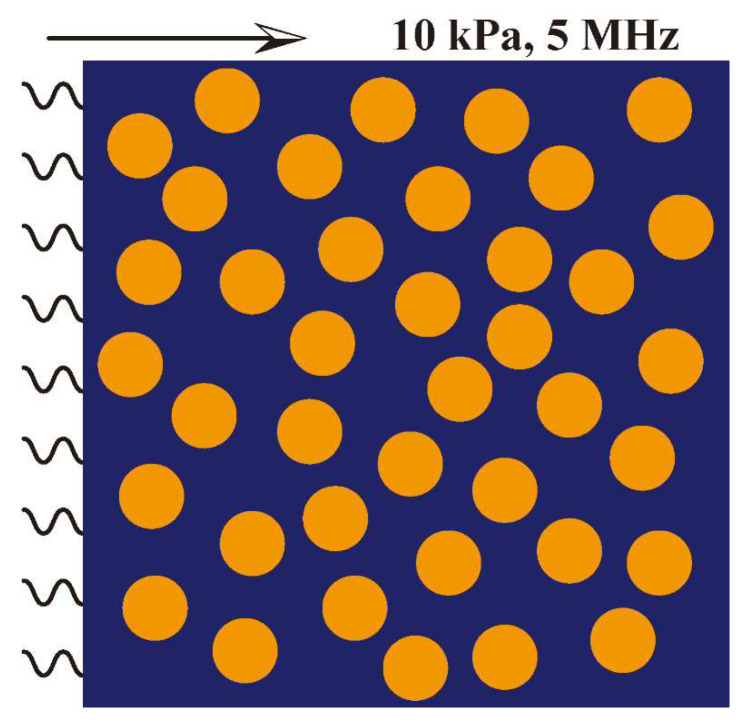
Typical Finite Element Model.

The behavior of elastic wave propagation in the Cu/PMMA composites was analyzed using finite element analysis software. The simulation entailed randomly arranging spherical Cu particles in a rectangular shape measuring 1 × 1 mm^2^ and filling the remaining space with PMMA, as depicted in Figure 2. A single period sinusoidal stress wave with a peak stress of 10 KPa and frequency of 5 MHz was loaded onto the left-hand boundary of the rectangle. To account for the attenuation of the Lamé waves, the attenuation coefficient of PMMA was added to the simulation according to the Rayleigh damping model. The experimentally derived attenuation coefficient of PMMA was 72 Np/m. The Cu particles were considered rigid owing to the substantial differences in the properties of Cu and PMMA. The parameters of the finite element simulation are summarized in Table 1.

The elastic wave velocity (c) and attenuation coefficient (α) were calculated using the following formulas:

(7) $$c=\frac{L}{T_{2}-T_{1}},$$

(8) $$\alpha =\frac{1}{L}ln\frac{A_{int}}{A_{out}},$$

## 3. Results and Discussion

### 3.1. Composites Structures

**Figure 3:**
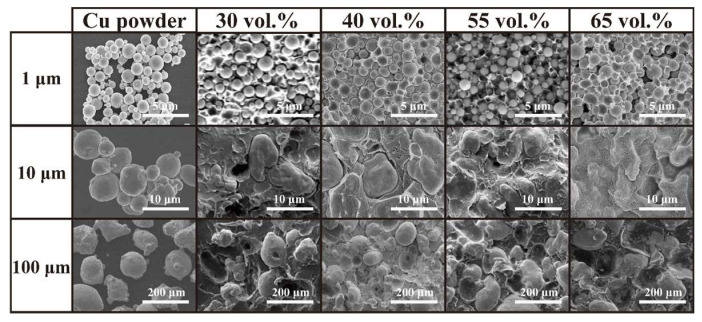
SEM images of Cu Powder and Cu/PMMA composites.

The results of the microstructure analysis revealed that all the Cu powders possessed a spherical shape, and the resulting Cu/PMMA composites exhibited a typical particle-filled composite structure, as depicted in Figure 3. The Cu powder was randomly and uniformly dispersed within the PMMA matrix without any significant flaws or voids, as shown in Figure S1. The actual Cu content of the Cu/PMMA composites listed in Table S1 was found to be within 5% of the expected content. This suggests that the preparation of the Cu/PMMA composites was successful and that the samples were homogeneous with a consistent filler distribution.

### 3.2. Elastic Wave Velocity and Attenuation

**Figure 4:**
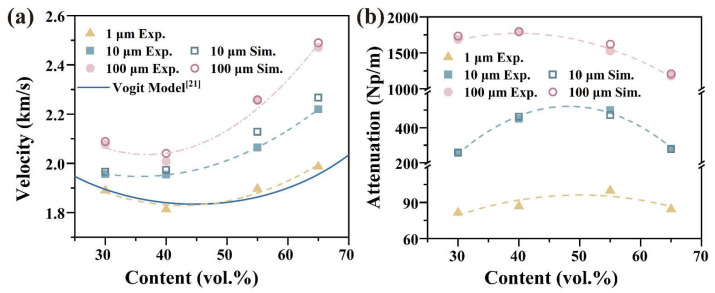
Elastic wave Propagation Behavior of Cu/PMMA composites: (**a**) Elastic wave velocity; (**b**) Elastic wave attenuation.

Figure 4 depicts the similarities between the simulation and experimental results for the elastic wave propagation behavior in the Cu/PMMA composites, which verifies the precision of both methods. Nonetheless, in some instances, the samples may not be entirely compact, leading to a slight difference between the simulation and experimental attenuation values. The elastic wave velocity and attenuation exhibit an inflection phenomenon for volume fractions of Cu particles ranging from 30 to 65%. This phenomenon has also been observed in PS/EPDM blends [31] and TiO_2_/PDMS [32]. The findings indicate that as the particle size of Cu powder increases, there was a growth in the wave velocity, in addition to an increase in the order of magnitude surge in the wave attenuation coefficient. For example, the wave velocity ranges from 1.89 km/s to 2.01 km/s in Cu/PMMA composites containing 30 vol. % of particles, and the wave attenuation coefficient ranges from 81.6 Np/m to 1678.8 Np/m as the particle size increases from 1 μm to 100 μm. With an increase in the particle size, the inflection point of the wave velocity and attenuation moves in the direction of the lower content.

### 3.3. Analysis of Wave Velocity and Attenuation

**Figure 5:**
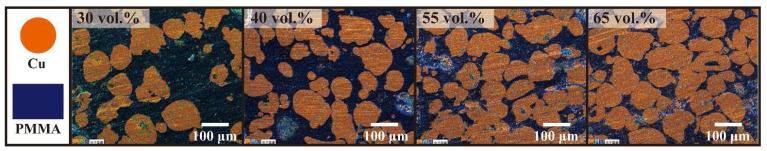
Distribution of 100 μm of Cu particles in the Cu/PMMA.

Figure 5 shows the distribution of Cu particles (orange areas) in the PMMA matrix (blue areas). For particle volumes less than 40 vol. %, the proximity of Cu particles decreases, resulting in an increase in the probability of elastic wave scattering between particles. Conversely, if the Cu particle content exceeds 55 vol. %, the overall velocity of the sample increases significantly due to the progressive rise in Cu content, which is considerably greater than that of PMMA.

The total attenuation of elastic waves in PPCs is a highly complex phenomenon that results from a combination of multiple factors. Because the matrix is viscoelastic, the total attenuation can be decomposed into matrix absorption, particle-independent scattering, and particle interaction. The total attenuation of elastic waves in particle-filled composites with high particle content can be estimated by applying the following formula:

(9) $$\alpha_{sum}=\alpha_{mat}+\alpha_{par}+\alpha_{int},$$

where $\alpha_{sum}$ is the total attenuation, $\alpha_{mat}$ is the matrix absorption, $\alpha_{par}$ is particle-independent scattering, and $\alpha_{int}$ is the particle interaction.

$\alpha_{mat}$ can be calculated by the following formula:

(10) $$\alpha_{mat}=\left(1-\emptyset_{par}\right)\alpha_{\mathrm{PMMA}},$$

The particle-independent scattering component can be calculated by examining the scattering of individual particles while disregarding their interactions within the composite. The objective of this study was to investigate the elastic solution of a sphere by considering a single particle in an infinite matrix.

**Figure 6:**
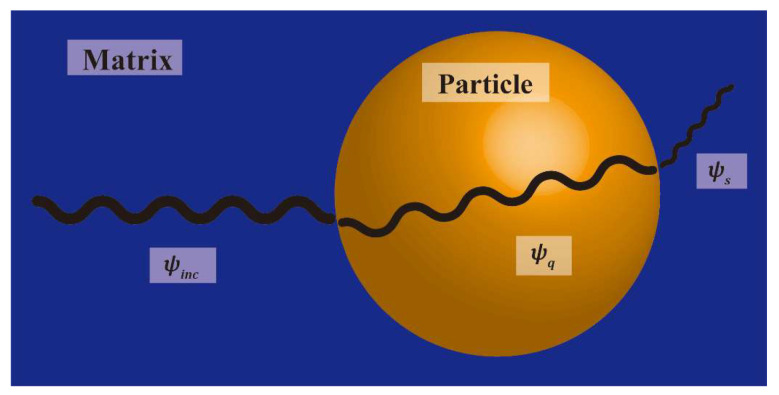
Schematic of elastic waves propagated by a single particle in PPC.

The study utilizes spherical coordinates, with the origin of the coordinate system set at the center of the spherical particle, as depicted in Figure 6. The displacement expressions for the external scattering wave and internal refraction wave of the spherical particle are as follows:

(11) $$\psi_{s}=\sum_{m=0}^{\infty }A_{m}h_{m}\left(k_{1}r\right)P_{m}\left(cos\theta \right),r\;>a,$$

(12) $$\psi_{q}=\sum_{m=0}^{\infty }B_{m}j_{m}\left(k_{2}r\right)P_{m}\left(cos\theta \right),r\;<a,$$

where ψ is the coordinate r and θ functions, and the subindices s and q are the scattered and refracted waves, respectively. $h_{m}$ is the third Bessel function, $j_{m}$ is the first Bessel function, $P_{m}$ is the m degree Legendre polynomial, and k is the elastic wave number.

$A_{m}$ and $B_{m}$ can be obtained using boundary conditions [40]. In actual calculations, m is taken from 0 to 50, and the scattering cross-section *γ* of a single-particle inclusion can be derived by incorporating the calculated results into the displacement and stress fields as follows:

(13) $$\gamma =4\pi \sum_{m=0}^{\infty }\frac{1}{2m+1}\left[{\left|A_{m}\right|}^{2}+m\left(m+1\right)\frac{k_{1}}{\kappa_{1}}{\left|B_{m}\right|}^{2}\right],$$

Particle independent attenuation, $\alpha_{par}$, can be obtained using the following formula:

(14) $$\alpha_{par}=\frac{1}{2}n_{s}\gamma^{par},$$

where *n_s_* is the number of particles.

**Figure 7:**
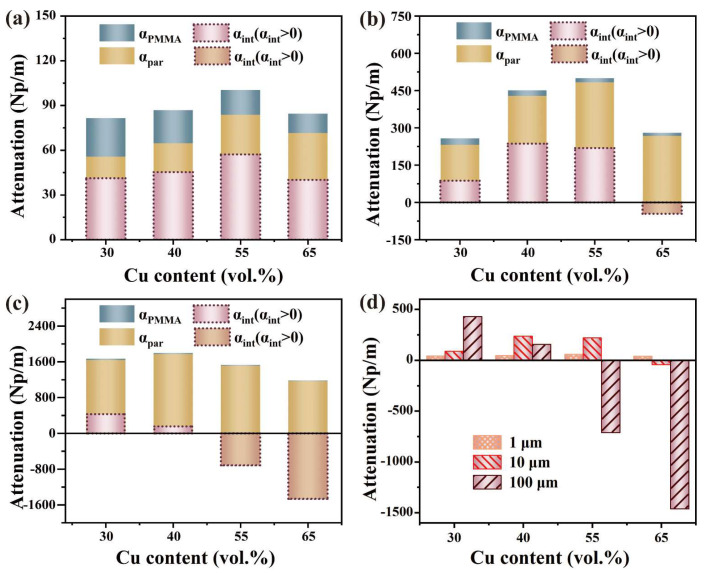
Each component of attenuation: (**a**) 1 μm Cu/PMMA; (**b**) 10 μm Cu/PMMA; (**c**) 100 μm Cu/PMMA; and (**d**) particle interaction of 1, 10, 100 μm Cu/PMMA.

The total attenuation of the elastic waves in the Cu/PMMA composites was decomposed into three parts, as shown in Figure 7. As the particle size increased, the proportion of $\alpha_{\mathrm{mat}}$ in the total attenuation decreased, with the ($\alpha_{par}+\alpha_{int}$) becoming the primary component of the total attenuation. Liu [41] also observed that scattering became the dominant mechanism of energy attenuation when the particle size was comparable to the elastic wavelength. In addition, $\alpha_{\mathrm{int}}$ changed as the particle size varied. At a particle size of 1 μm, $\alpha_{\mathrm{int}}$ initially increased, then decreased, and finally remained positive. At a particle size of 10 μm, $\alpha_{\mathrm{int}}$ initially increased and then decreased, and the particle interaction became negative at 65 vol. %. At a particle size of 100 μm, $\alpha_{\mathrm{int}}$ was positive with 30 and 40 vol. % contents, but negative with 55 and 65 vol. % contents. $\alpha_{\mathrm{mat}}$ increases linearly with the increase in Cu content, yet it always constitutes only a minor portion of the total attenuation. $\alpha_{par}$ increases with both the Cu content and particle size. $\alpha_{int}$ initially increased and then decreased as the Cu content increased, leading to an inflection point in the total attenuation.

**Figure 8:**
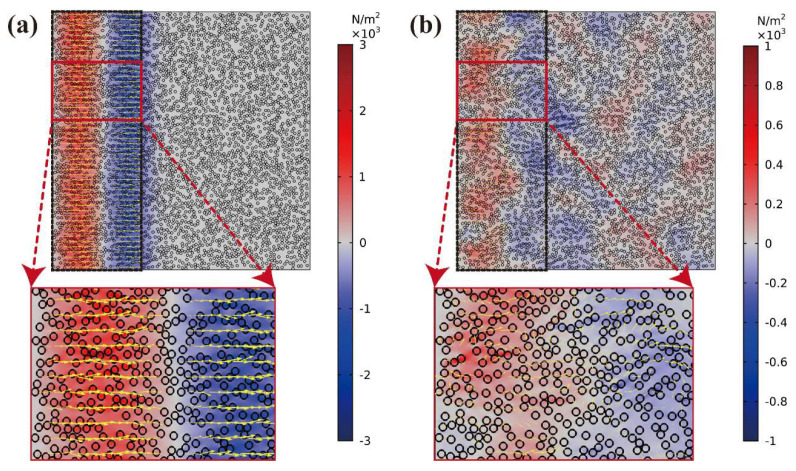
Stress distribution of elastic wave propagating behavior in Cu/PMMA composites: (**a**) At 0.4 μs; (**b**) At 1.2 μs.

At 0.4 μs, the stress distribution of the elastic wave propagation in the sample is shown in Figure 8a. The stress direction aligned almost perfectly with the initial loading stress wave, and the influence of the particle interaction was negligible. The aftershock occurred after an initial stress wave loading of 1.2 μs, as shown in Figure 8b. The direction of stress propagation in the aftershocks caused by particle interactions is disorderly. The blue and red colors represent tensile and compressive waves, respectively, and the yellow arrows indicate the direction of stress propagation. To emphasize the direction of stress propagation, the arrows in Figure 8a,b were enlarged because the intensity of the aftershock was too small. Hence, the arrows can only represent the direction of the stress propagation wave.

**Figure 9:**
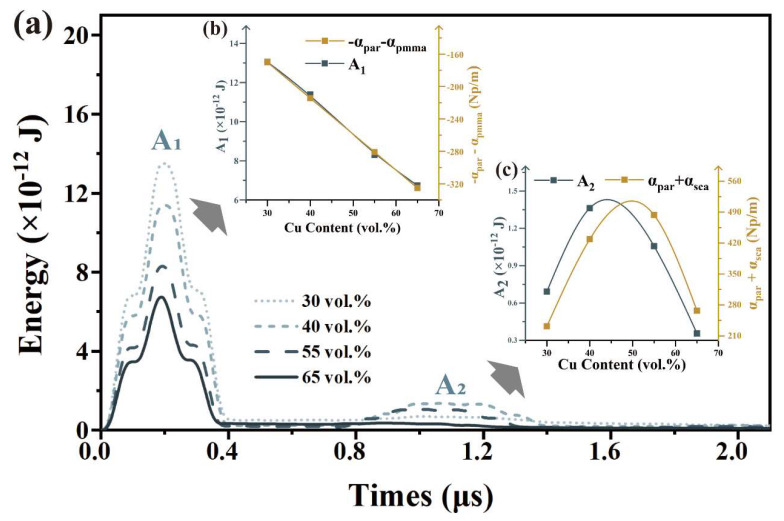
Analysis of mechanical energy after wave of 10 μm Cu/PMMA: (**a**) Mechanical energy intensity; (**b**) Height of the first peak(A_1_) and $\left(-\alpha_{mat}-\alpha_{par}\right)$; and (**c**) Height of the second peak(A_2_) and ($\alpha_{par}+\alpha_{sca}$).

The mechanical energy history of the leftmost third of the sample, which is represented by the black box (3.33 mm × 1 mm), is illustrated. As shown in Figure 9a, the mechanical energy intensity within the black dotted box sample is illustrated by the first peak (A_1_) occurring in 0–0.4 μs and the second peak (A_2_) occurring in 0.8–1.4 μs. A_1_ represents the initial input elastic wave intensity attenuated by matrix absorption and single-particle scattering ($-\alpha_{mat}-\alpha_{par})$. At the initial stage when the elastic wave has just entered the sample, the primary cause of its attenuation is absorption by the matrix and particle-independent scattering. The caused by particle interactions can be neglected at this point.

The heights of the first peaks (A_1_) and the $\left(-\alpha_{mat}-\alpha_{par}\right)$ decreased linearly with increasing particle content, as shown in Figure 9b. The second peak (A_2_) is an aftershock caused by independent particle scattering and particle interactions behind the wavefront [42]. Figure 9c indicates that the height of the second peak is consistent with the changing trend of single-particle scattering and particle interaction ($\alpha_{par}+\alpha_{sca}$), with both initially increasing and then decreasing as the particle content increases.

**Figure 10:**
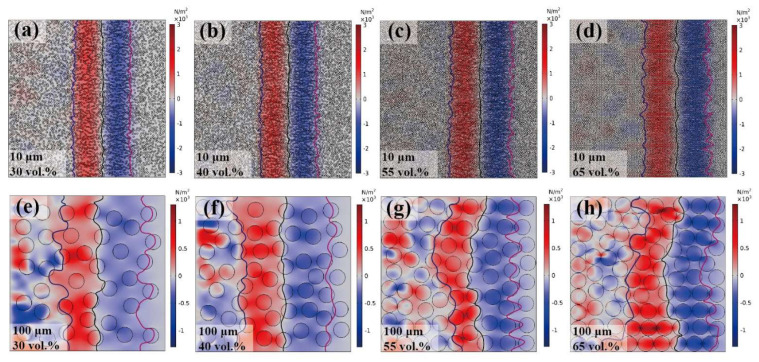
Stress distribution of elastic wave propagating behavior in Cu/PMMA composites: (**a**–**d**) particle size of 10 μm; (**e**–**h**) particle size of 100 μm.

The wavefront consisted of compressive and tensile waves caused by a sinusoidal incident wave, as illustrated in Figure 10. When the particle size was held constant, the width of the elastic wavefront remained unchanged regardless of the variation in the filling particle content. However, the wavefront width was observed to increase with increasing particle size. For instance, in a Cu/PMMA composite with a particle size of 10 μm, the width of the elastic wavefront is approximately 400 μm, whereas that of the Cu/PMMA composite with a particle size of 100 μm is approximately 800 μm. Rauls [43] reported a similar phenomenon in the case of shock waves, where the wavefront width is directly proportional to the particle size.

**Figure 11:**
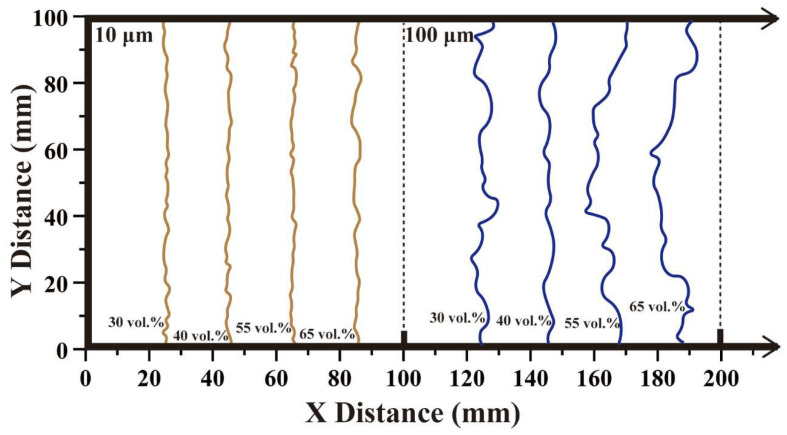
Central line of the wavefront from simulations after elastic wave load for 40–60% volume fraction of 10 μm (**left**) and 100 μm (**right**) in Cu/PMMA.

Figure 11 depicts the central line of the wavefront in Cu/PMMA composites at 0.4 μs after the elastic wave load with zero stress value. Owing to the inherent randomness of the composite geometry, the ripple period is not constant. When the composites were composed of tiny particles, the magnitude of the surface ripples and peak-to-peak distance was small, as shown in Figure 11 (left) for the 10 μm particles. The rippling of the central line had an amplitude of approximately 20 μm. In the 100 μm particle size case shown in Figure 11 (right), the amplitude of the main line is approximately 100 μm. An increase in the particle size has a significant impact on the degree of disorder in wavefront ripples.

Thus, the dominant factor contributing to attenuation in PPC is ($\alpha_{par}+\alpha_{sca}$), which initially increases and then decreases as the Cu content increases, resulting in the inflection phenomenon observed in the attenuation of elastic waves. Furthermore, it is evident that the particle size can significantly alter the elastic wave velocity, attenuation, and wavefront characteristics by affecting the elastic wave propagation behavior.

## 4. Conclusions

In this investigation, Cu/PMMA composites were prepared using particles with sizes ranging from 1 to 100 μm and contents varying from 30 to 65 vol %. The pulse-echo method and FEM simulation were employed to examine the impact of particle size and content on the elastic wave propagation behavior of the PPC. As the particle content increased to 65 vol. %, an inflection phenomenon was observed in the elastic wave velocity and attenuation of the PPC. Based on the classical elastic wave scattering theory, the attenuation of the elastic wave in the high-particle-content PPC is divided into matrix absorption ($\alpha_{mat}$), particle-independent scattering ($\alpha_{par}$), and particle interaction ($\alpha_{int}$). It was found that ($\alpha_{par}+\alpha_{sca}$) is the main factor causing the inflection phenomenon of elastic wave attenuation in the PPC. Moreover, increasing the particle size significantly affects the elastic wave velocity, attenuation, and width of the wavefront characteristics.

To summarize, the structure has a profound impact on the elastic wave-absorbing properties of PCC by influencing the elastic wave propagation and attenuation characteristics. This study elucidated the mechanism of elastic wave propagation behavior in high particle content PPCs and provided a new way for the design and application of wave-absorbing composites. In future studies, more structural factors, such as particle size distribution, particle size number, and particle distribution pattern, can be taken into account in the structural design and application of absorbent PPCs.

## Figures and Tables

**Table 1:** Finite element simulation parameters.

	c_l_	c_s_
Cu	4763	2318
PMMA	2740	1372

## Data Availability

Data available in a publicly accessible repository.

## Supplementary Materials

The following supporting information can be downloaded at: https://www.mdpi.com/article/10.3390/polym15071680/s1, Figure S1: C.T. Scan results for Cu/PMMA composites; Table S1: Preparation and actual content of Cu/PMMA composites.
