# Supplementary material for: Influence on Elastic Wave Propagation Behavior in Polymers Composites: An Analysis of Inflection Phenomena
Source: Polymers (Basel). 2023 Mar 28;15(7):1680. doi: 10.3390/polym15071680 (PMC10096520; doi:10.3390/polym15071680)
Supplement: Supplementary file 1 [file polymers-15-01680-s001.zip › polymers-2288083-supplementary.pdf]

Supplementary Materials:

Influence on elastic wave propagation behavior in polymers composites: An analysis of inflection phenomena

Guoqiang Luo [1, 2], Pu Cheng [2], Yin Yu [3], Xiangwei Geng [2], Yue Zhao [2], Yulong Xia [2], Ruizhi Zhang [2,3,*], Qiang Shen [2]

[1] Chaozhou Branch of Chemistry and Chemical Engineering Guangdong Laboratory, Chaozhou 521000, China
[2] State Key Lab of Advanced Technology for Materials Synthesis and Processing, Wuhan University of Technology, Wuhan 430070, China
[3] National Key Laboratory of Shock Wave and Detonation Physics, Institute of Fluid Physics, China Academy of Engineering Physics, Mianyang 621900, China
* Correspondence: zhangrz1991@gmail.com; Tel.: +86-130-3514-9005

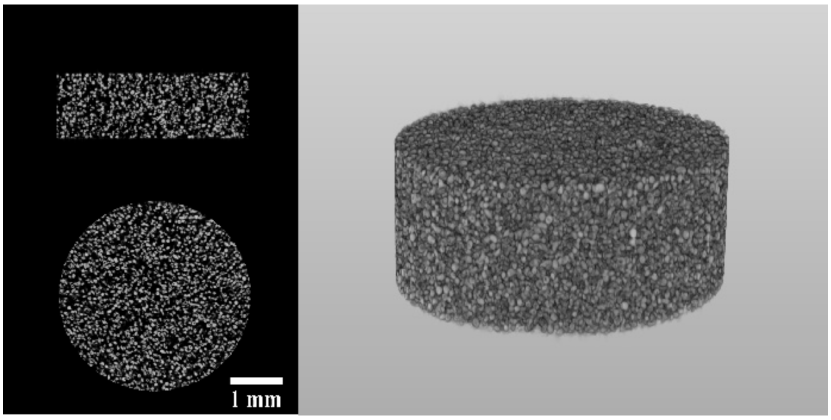

Figure S1. C.T. Scan results for Cu/PMMA composites

Table S1. Preparation and actual content of Cu/PMMA composites

Design content (vol. %)	Particle size (μm)	Mixing temperature (℃)	Mixing Time (min)	Hot pressing temperature (℃)	Hot pressing times (min)	Actual content (vol. %)	Error value (%)
	1					29.27	2.43
30	10	210	20	200	70	29.77	0.77
	100					29.14	2.87
	1					40.12	-0.30
40	10	210	20	200	70	40.71	-1.78
	100					41.28	-3.20
	1					53.74	2.29
55	10	210	20	200	70	54.22	1.42
	100					53.76	2.25
	1					65.22	-0.34
65	10	220	20	210	100	66.78	-2.74
	100					67.01	-3.09
